# Developmental trajectories in laryngeal anatomy of California mice (*Peromyscus californicus*)

**DOI:** 10.1098/rsos.251029

**Published:** 2025-10-08

**Authors:** Mia Sherwood, Mackenzie Kaup, Karen L. Baab, Tobias Riede

**Affiliations:** ^1^ Department of Physiology, College of Graduate Studies, Midwestern University, Glendale, AZ 85308, USA; ^2^ Department of Anatomy, College of Graduate Studies, Midwestern University, Glendale, AZ 85308, USA

**Keywords:** rodents, vocal production, voice, geometric morphometrics

## Abstract

The mammalian larynx is a complex structure of mixed embryological origin, with its evolutionary diversification and its form–function relationship of interest to biologists and clinicians. This study compared the size and shape of the laryngeal cartilaginous framework and airway in two phylogenetically distant mouse species, the California mouse and House mouse, with distinct vocal behaviours. Using three-dimensional imaging and geometric morphometrics, we analysed ontogenetic shape changes from birth to old age to assess species differences, developmental trajectories and integration of cartilage shape. While statistically significant species-specific differences were found, they were minor, and neither species exhibited sexual dimorphism in laryngeal shape or size. A pronounced ontogenetic change in the relative size of the ventral pouch was observed in California mice but not in House mice. Shape changes from neonatal to adult stages were largely conserved across species, with the notable exception of the arytenoid cartilage, which exhibited divergent postnatal trajectories and integration patterns. Despite differences in vocal behaviour and phylogenetic distance, overall laryngeal morphology was remarkably similar. These findings highlight the need to consider additional selective pressures beyond vocal function in shaping laryngeal anatomy across rodents.

## 1. Introduction

Rodents have undergone extensive radiation and now account for over 50% of known mammalian species [1]. Most rodent species live in social groups [2] and rely on vocal communication [3,4]. Diverse vocal behaviour may be tied to structural variation in the vocal organ [5–7], and/or to differences in the neural circuitry controlling vocal movements [8]. However, it is unknown whether the diversity in vocal behaviour among rodents is linked to morphological variation in their vocal organs, and if morphological variation was shaped by evolutionary pressures on vocal communication. This study aimed to describe the development and integration of the larynx in California mice (*Peromyscus californicus*) and House mice (*Mus musculus*) to improve our understanding of its function and to provide a foundation for comparative studies, offering insights into broader patterns of form and function across all rodents.

The form of the mammalian larynx impacts its function as a valve, protecting the respiratory tract during breathing and swallowing, and as a vocal organ. It houses the vocal folds, which enable voice production. In rodents, it also houses the ventral pouch, which facilitates ultrasonic whistling, a vocal behaviour unique to this clade [9,10]. The mammalian larynx is a complex structure consisting of a cartilaginous framework, multiple sets of intrinsic skeletal muscles, ligaments and an epithelial lining. The shape of individual elements, such as the four main cartilages, varies among mammal species [11–13], exhibits sexual dimorphism in some species [14,15], and changes in shape and size throughout an individual’s life [16,17]. Certain shape differences within and among species are evidently related to their vocal function. For example, the greater size of the larynx in male humans houses longer vocal folds, which facilitate slower vibration rates and consequently lower-pitched voices compared with females [18]. Even more nuanced shape differences between professional human singers and non-singers have been hypothesized [19]. In rodents, there are few anatomical studies on overall laryngeal shape and size available [11,13], but the current literature hints at considerable variation among and within species [16,20]. To fill this knowledge gap, we contrast existing data on larynx shape and size in House mice [16,21] with new data from the California mouse.

The two mouse species vary markedly in geographic, ecological and behavioural patterns (e.g. [22–24]). The House mouse tends to live in large groups and exhibits high behavioural plasticity, ranging from strongly territorial behaviour to gregarious social groups for at least part of the year [25]. The California mouse lives in monogamous pairs that defend a territory and exhibit biparental care [26,27]. House mice have a much shorter generation time (9–11 weeks) compared with California mice (20–25 weeks in captivity). House mice are prolific breeders with large litters (5 to 10 pups) and a gestation period of 21 days. They reach sexual maturity at 6−8 weeks of age. By contrast, California mice have a gestation period of 28 days, reach sexual maturity at around 150 days of age (in captivity), and produce smaller litters ranging from 1 to 4 pups (average 1.7 pups/litter [28]). Parental investment in each pup is considerably higher in California mice than in House mice [29,30]. Litter size affects parental investment, which in turn impacts foetal and postnatal growth rates [31,32]. The House mouse (CD1 outbred stock) has been bred for research purposes since 1926 [33], whereas *Peromyscus californicus* has been in captivity since 1976 [34].

Both species rely on vocal communication [35–38]. Both species employ two mechanisms of vocal production, airflow-induced vocal fold vibration and high-frequency whistling [9,10,39] but the relative rate at which the two mechanisms are employed are different. California mice produce long-distance calls by airflow-induced vocal fold vibration [40,41]. Long-distance calls serve as signals to mark a territory and communicate between established pairs over distances up to 100 m which is remarkable for a small ground-dwelling rodent [42]. Sounds produced by airflow-induced vocal fold vibration are of higher intensity and place the larynx under much larger stresses than whistling [43,44]. By contrast, House mice predominantly exhibit ultrasonic whistling. Those high-frequency sounds tend to carry only over short distances [45]. We investigated whether this difference in vocal behaviour is associated with size or shape differences of the vocal organ, focusing on the laryngeal cartilaginous framework and airway.

The two muroid mouse species belong to different families, Muridae and Cricetidae, respectively, which shared a common ancestor more than 10 million years ago [46] but share a similar body size. The larynx of House mice does not exhibit sexual dimorphism in size or shape, all four laryngeal cartilages scale with negative allometry on body size, and the cartilaginous framework of the larynx changes in shape throughout life [16,17,21]. The shape changes of laryngeal cartilages are associated with remodelling of laryngeal soft tissue, and intra-laryngeal airway [21]. California mouse larynx morphology is undocumented. Investigations of these two phylogenetically distant mouse species in larynx shapes and postnatal developmental trajectories will potentially identify key drivers of laryngeal variation. We included high-dimensional shape analysis of three-dimensional reconstructions of the laryngeal cartilages and airways generated from contrast-enhanced micro-CT data in mice aged postnatal day 1 to old age.

Finally, we compared morphological integration between the two species. The question of morphological integration is interesting because the larynx is a complex anatomical structure that originates embryologically from multiple germlayers [47,48]. Trait variation among individual components of the larynx or how the different cartilages co-evolved is very little understood. Morphological integration refers to how parts of an organism covary such that changes in a single feature may induce changes in other traits. This coordination of trait variance, produced by developmental mechanisms, for example, can act to maintain functional competence [49,50]. In the case of the larynx, a high magnitude of integration could support precise control of the morphology of individual cartilages (and their associated soft tissues) to maintain the exact morphological configuration necessary to control respiration and sound production.

## 2. Methods

California mice (*Peromyscus californicus*) were bred at Midwestern University. The breeding colony was established in 2019 with eight animals acquired from the Peromyscus Genetic Stock Center. A total of 34 animals (16 females; 18 males) of known age were included in the study (postnatal days 2 to 1500) (see supplementary material for details). Animal care procedures were per guidelines established by the American Society of Mammalogists [51]. Procedures were approved by the Institutional Animal Care and Use Committee at Midwestern University, Glendale, Arizona (protocol #4112).

A similar dataset of 30 larynges from *Mus musculus* (CD1 mice), investigated previously [16,21], was included for comparison. The dataset consists of five age groups (postnatal days: 2, 21, 90, 365, 720). In each age group, 3 males and 3 females were included.

### 2.1. Tissue collection and three-dimensional reconstruction

Body mass and femur length were measured from each animal. Body mass is dependent on skeletal mass, lean mass and fat mass, which can vary independently. Long bone length is more closely tied to skeletal size [52]. We initially observed that the California mouse and House mouse samples were similar in body mass and tested whether this was also true for skeletal size.

Larynx tissue was carefully dissected from animals euthanized with Ketamine/Xylacine and perfused with 0.9% saline solution followed by 10% buffered formalin (SF 100-4, Fisher Scientific). Excised tissue was fixed in 10% buffered formalin for 12 h and then transferred to 99% ethanol. The tissue was stained with phosphotungstic acid (Sigma-Aldrich, #79690) for 2 to 5 days. Stabilized in polystyrene craft foam, the tissue was scanned in air with a Skyscan 1172 (Bruker-microCT) (59-kV source voltage; 167-µA intensity; angular increment of 0.4 over a 180 rotation; vocal size: 4.5 to 5.4 µm per pixel). AVIZO software (version Lite 9.0.1) was used to segment laryngeal cartilages and laryngeal airway. Segmentation was performed manually outlining the boundary between cartilaginous elements and surrounding soft tissue, and between the airway and the soft tissue. The ventral pouch was segmented separately by outlining the boundary between the airway and the soft tissue on its ventral and lateral sides. The dorsal boundary was determined by drawing a straight line between the alar edge and the ventral insertion of the vocal folds. Surface renditions in stereolithography format of all specimens are available on Morphobank [53], project 5517. The three-dimensional reconstruction files for *Mus musculus* were available through Morphobank projects 3635 and 4018 [16,21].

### 2.2. Shape analysis

The variability of laryngeal morphology between species and the absence of homologous anatomical landmarks has previously hindered robust comparison of laryngeal shape using linear measurements. We used high-density shape analysis (geometric morphometrics) following an approach outlined in Riede *et al.* [16] to comprehensively capture larynx morphology among age groups or between species.

**Figure 1.**
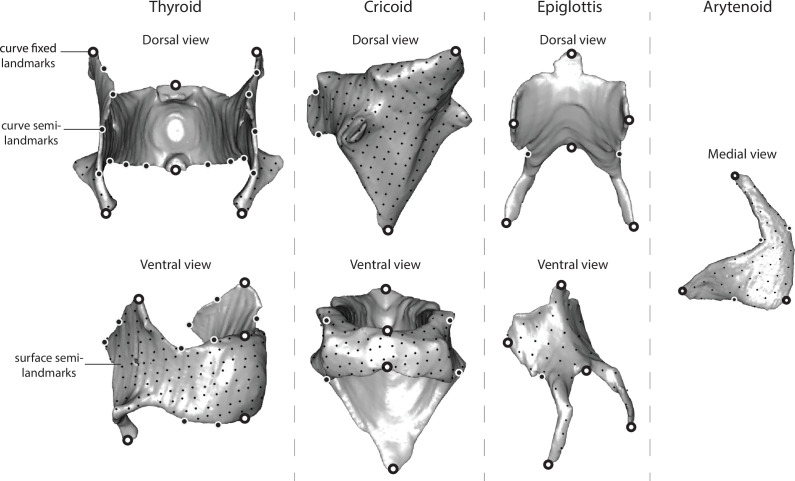
Curve and surface landmarks on four larynx cartilages from a California mouse. ‘Curve landmarks’ (large circles) were placed manually, and ‘surface landmarks’ (small circles) were initially identified on a single specimen to build a template. These landmarks were transferred to the remainder of the sample using geomorph’s *build-template* function.

Three-dimensional landmarks and curve/surface semilandmarks (hereafter referred to as landmarks) were used to quantify the shape of four cartilages (thyroid cartilage, cricoid cartilage, arytenoid cartilage, epiglottis) (figure 1; curve landmarks defined in table 1) for members of both species. The minimum number of landmarks necessary to fully capture shape variation was determined for each cartilage (thyroid cartilage: 100; cricoid cartilage: 100; arytenoid cartilage: 50; epiglottis: 50) based on a sensitivity analysis presented by Borgard *et al*. [20]. Landmarks were placed using the geomorph package in R 4.0.7 [54].

**Table 1.** Explanation of curve landmarks that were manually placed on each cartilage. Surface landmarks were additionally placed on each cartilage.

	thyroid cartilage
1	posterior most point on rostral margin of left cranial process
2	midline point on cranial margin
3	posterior most point on cranial margin of right cranial process
4	posterior most point on caudal margin of right caudal process
5	midline point on caudal margin
6	midline point on caudal margin

	cricoid cartilage
1	posterior most point on cranial margin
2	most left point on cranial margin
3	anterior most point on cranial margin
4	most right point on cranial margin
5	posterior most point on caudal margin
6	most left point on caudal margin
7	anterior most point on caudal margin
8	most right point on caudal margin

	arytenoid cartilage
1	most distal point on cranial process
2	most distal point on vocal process
3	most distal point on muscular process

	epiglottis
1	most cranial point along midsagittal line
2	most left point on lateral margin
3	most caudal point along midsagittal line
4	most right point on lateral margin

Next, we performed a partial Generalized Procrustes Analysis (GPA) using the geomorph package in R, with sliding semi-landmarks to minimize bending energy. This approach aligns with standard practices in geometric morphometrics and ensures reproducibility [55,56]. Tangent space coordinates obtained after Generalized Procrustes Analysis were used for statistical analysis. We employed principal components analyses (PCAs) to reduce the highly dimensional dataset to a smaller number of dimensions (PC axes) that summarize the main patterns of shape variation. One or more dimensions may align with age and size variation in the sample, but this is not a given. The PCA was performed on the superimposed landmark data.

To illustrate anatomical shape differences with age and size, a specimen close to the mean shape was identified. The selected specimen was warped to the mean shape using the ‘warpRef-Mesh’ function in geomorph. Shape was regressed onto age to produce loadings (regression coefficients). The loadings of the respective variables were then added and subtracted from the mean shape to produce the shapes at the positive and negative ends of the axis.

Five dimensions were also measured from each specimen. Centroid size, computed as the square root of the sum of squared distances of each landmark from the centroid, captures overall size of each cartilage [56,57]. The dorso-ventral and latero-lateral diameters of the airway measured at the caudal end of the cricoid cartilage using the linear measure tool in AVIZO were taken as proxies for airway size. The membranous vocal fold length was measured between the vocal process of the arytenoid cartilage and the ipsilateral protuberance on the medial surface of the thyroid cartilage, also using the linear measure tool in AVIZO. Vocal fold length is an important determinant of fundamental frequency in vocalizations produced by airflow-induced vocal fold vibrations [58]. The fifth parameter was ventral pouch volume. First, the ventral pouch was three-dimensionally reconstructed separately. Then the three-dimensional viewer plugin of FIJI-ImageJ software [59] was used to estimate the volume of the ventral pouch using the ‘*measure volume*’ function. Ventral pouch geometry has been suggested as a critical determinant of fundamental frequency of rodent ultrasonic whistles [9,10].

### 2.3. Statistical analysis

The relationship between body size and laryngeal size was analysed using linear mixed-effects models, with log-transformed body mass (logBM) as a fixed effect and individual identity included as a random intercept to account for repeated measures within individuals. Additional fixed effects included sex and its interaction with body mass. Scaling relationships for femur length, vocal fold length, airway diameter and centroid size were evaluated by comparing observed slopes to the isometric expectation (body mass^0.33^), i.e. body mass and all linear measures were log-transformed, and the scaling relationship was evaluated based on a theoretical slope of 0.33.

A multivariate analysis of variance was performed to determine whether there were statistically significant differences in cartilage shape related to sex or age. Linear mixed effect models (type II) were fitted to the high-dimensional dataset of all principal components. We first run the full model including sex, age, a size variable (centroid size, femur length, body mass) and their pairwise interactions for each of the four cartilages. Variation inflation factors (VIF) were used to avoid multicollinearity and optimize the models. VIF were large (>10) for age and any size variable when included in the same model, indicating multicollinearity between age and size (table 2). Therefore, only age was used in a more restricted model. We did model comparisons between the full model and restricted models based on the variables that contributed most strongly and only presented the best-supported model. Sex, age and their two-way interactions were sequentially introduced into the linear model and statistical significance was assessed via a randomized residual permutation procedure using the RRPP package for R (vers. 2.0.0.) [60].

**Table 2.** Variance Inflation Factors (VIF) for sex, age and three size variables in multilinear models exploring sources of shape variation. The VIF values assess multicollinearity in multilinear models used to investigate shape variation, with values above 10 indicating significant multicollinearity.

variable	thyroid cartilage	cricoid cartilage	arytenoid cartilage	epiglottis
sex	1.1	1.1	1.1	1.1
age	16.0	18.2	16.0	16.3
femur length	48.2	46.9	49.2	61.6
body mass	37.5	34.6	34.8	31.5
centroid size	14.7	9.5	12.5	11.7

We used two approaches for species comparison. We present the results of the size and shape analyses of the laryngeal cartilages in California mice and discuss how they compare with results of identical analyses published previously for the House mice [16,21]. Cartilage shape was compared between the two species by warping the average cartilage shape into the respective species average illustrated by thin-plate-spline diagrams. Linear mixed effect models were used to investigate the effect of species on larynx shape.

Next, trajectory analysis was conducted to evaluate the path distance and direction of shape changes for each of the four laryngeal cartilages. Shape trajectories were calculated for five age groups (postnatal days 2−20, 21−49, 50−100, 101−499, 500−2000), representing stages from neonates to older adults. For both House mice (MM) and California mice (PC), *distance* values represent the total length of each trajectory, indicating the magnitude of shape change between the start and end points. The statistical test compares these trajectory lengths between the two species to assess differences in the magnitude of shape change. Trajectory *direction* refers to the vector along which shape changes occur, representing the orientation of the trajectory in morphospace. The test evaluates whether the pattern of shape changes differs between the species by measuring the angle between them. The statistical analysis of developmental trajectories was computed using the ‘trajectory.analysis’ function in the RRPP package for R [61]. A two-sample testing procedure for comparing slopes of developmental trajectories between the two species is provided using code in the R-package geomorph [62,63].

Magnitude of integration was quantified by the relative eigenvalue variance. Eigenvalues reflect the amount of variance captured by each eigenvector (principal component) determined from a covariance matrix, and relative eigenvalue variance reflects the eccentricity of variation along different dimensions in the morphospace of each cartilage. Relative eigenvalue variance (*V*_*rel*_) was estimated using equation (2.1) (after [62,64]):

(2.1) $$V_{rel}=\;\frac{\int_{i=1}^{p}{\left(\lambda_{i}-\lambda \right)}^{2}}{p\left(p-1\right)\lambda^{2}}$$

where *p* is the number of variables, *λ*_*i*_ is the *i*th eigenvalue of the covariance matrix and *λ* is the average of the eigenvalues. Thus, a high proportion of trait variance being concentrated onto a small number of dimensions (high eccentricity; values close to one) indicates strong integration, whereas a pattern of trait variance that is spread across many dimensions (low eccentricity; values close to zero) reflects weak integration. Relative eigenvalue dispersion is reported together with cumulative eigenvalues and proportion of variance on each eigenvector. A two-sample testing procedure for comparing the strength of integration between the two species is provided using code in the R-package geomorph (Adams *et al*. 2022; [62]).

## 3. Results

### 3.1. Size of laryngeal cartilages

**Figure 2.**
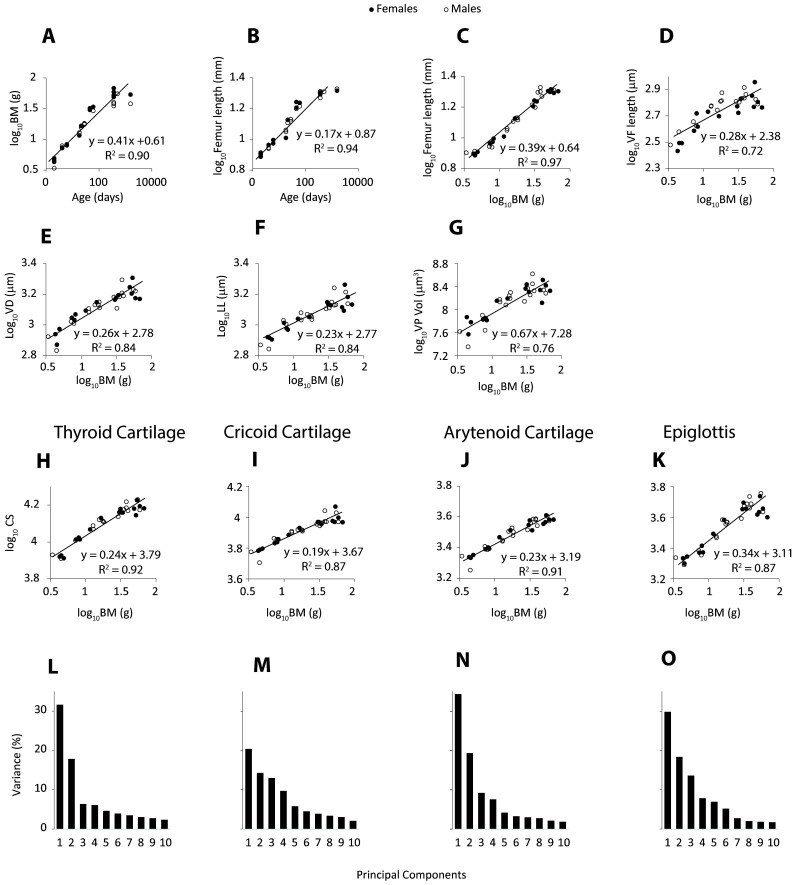
Size and shape of larynx in California mice. Animals ranged in age between postnatal day 2 and about 4 years. A total of 38 animals were included. Body mass (A) and femur length (B) increase with age (Note *Age* axis is logarithmic). Femur length (C), vocal fold length (VFL; D), airway dimensions (dorso-ventral (VD) and latero-lateral (LL) diameters of the airway measured at the caudal end of the cricoid cartilage; (E,F), and ventral pouch volume (G) are tightly associated with body mass, similarly in males and females. (H**–**K): The size of each of the four cartilages (expressed as centroid size, CS) scales allometrically with body mass. (L–O): A substantial portion of the shape variation is captured by the first few principal components capturing shape.

**Figure 3.**
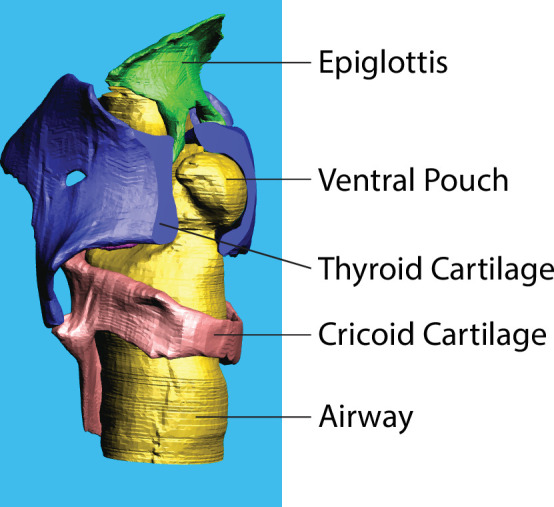
Three-dimensional representations of a California mouse larynx. The thyroid cartilage is ventrally sliced open to allow a view of the ventral pouch which is large and sphere-like in pups and flattens somewhat with age.

Ranges and test results for sexual differences and scaling on body mass are presented in table 3. At 1 year of age, California mice weighed 50 to 60 g. The largest femur length of about 21 mm was reached at the same age (figure 2A,B). Femur length scaled with positive allometry on body mass (figure 2C). Ventro-dorsal and latero-lateral airway diameter and vocal fold length scaled with negative allometry on body mass (figure 2D–F). Ventral pouch volume scaled with positive allometry (figures 2G and 3). None of the variables were sexually dimorphic (table 3).

**Table 3.** Ranges and test results for anatomical measurements in the California mouse sample. Age ranged between postnatal day 2 and 1500. To assess allometry, variables were log-transformed and regressed against log-transformed body weight. Regression slopes (variable b) greater than 0.33 indicate positive allometry relative to body growth, while slopes less than 0.33 indicate negative allometry (see also figure 2C-K). Centroid size, CS; Vocal fold length, VFL.

variable	*N*	range	sexual dimorphism	allometry
body mass (g)	34	3.4–68.0	*F* = 1.2; *p* = 0.362	
femur length (mm)	34	7.7–21.3	*F* = 0.38; *p* = 0.54	*b* = 0.39; *p* < 0.001
airway diameter (µm) ventro-dorsal latero-lateral	34	682–2028 698–1829	*F* = 0.59; *p* = 0.45 *F* = 0.34; *p* = 0.57	*b* = 0.26; *p* < 0.001 *b* = 0.23; *p* < 0.001
VFL (µm)	34	271–905	*F* = 0.14; *p* = 0.71	*b* = 0.28; *p* < 0.001
ventral pouch volume (mm^3^)	34	0.022–0.42	*F* = 1.67; *p* = 0.20	*b* = 0.67; *p* < 0.001
CS thyroid cartilage (*10^3^)	34	5.9–11.7	*F* = 0.14; *p* = 0.71	*b* = 0.24; *p* < 0.001
CS cricoid cartilage (*10^3^)	34	8.1–16.9	*F* = 0.01; *p* = 0.91	*b* = 0.19; *p* < 0.001
CS arytenoid cartilage (*10^3^)	34	1.8–4.1	*F* = 0.06; *p* = 0.81	*b* = 0.24; *p* < 0.001
CS epiglottis (*10^3^)	34	1.9–5.7	*F* = 0.30; *p* = 0.59	*b* = 0.34; *p* < 0.001

Centroid size of three laryngeal cartilages scaled with negative allometry on body mass (figure 2H–K). The epiglottis scaled isometrically on body mass (figure 2K). None of the four cartilages showed a sexually dimorphic development in size.

### 3.2. The effect of sex and age on laryngeal cartilage shape

Supplementary material, video 1 shows three-dimensional surface renderings of laryngeal cartilages of a 2-day-old and a 4-year-old male, indicating how the shape of four laryngeal cartilages changed with age. The first two shape axes accounted for 49.3%, 43.1%, 43.8% and 51.5% of total shape variation in the thyroid, cricoid, arytenoid and epiglottis cartilages, respectively (figure 2L–O).

We present a limited model which includes sex, age and their interaction (tables 4–7). Given strong collinearity of age and size variables, the size variables (body mass, femur length, centroid size) have a similar relationship with shape as that described for age. Corresponding results for House mice are reported in supplementary material, tables S1–S4.

**Table 4.** Results of the Procrustes ANOVA testing the effects of sex and age on shapes of four cartilages of *Peromyscus californicus* mice. The table shows the degrees of freedom (Df), sum of squares (SS), mean square (MS), Rsq = Coefficient of determination, *F*-statistic, *Z*-score and *p*-values for each factor. Statistically significant effects (*p < 0.05; **p < 0.01; ***p < 0.001) are indicated.

	Df	SS	MS	Rsq	*F*-score	*Z*-score	Pr (>F)	Signif.
				thyroid	cartilage			
sex	1	0.0043	0.0043	0.0170	0.7904	−0.9473	0.832	
log(age)	1	0.0378	0.0378	0.1499	6.3619	3.5115	0.001	***
sex * log(age)	1	0.0094	0.0094	0.0375	1.4161	1.0743	0.144	
residuals	30	0.2003	0.0067	0.7940				
total	33	0.2523						

				cricoid	cartilage			
sex	1	0.0067	0.0067	0.0264	0.927	0.0524	0.529	
log(age)	1	0.0220	0.0221	0.0868	3.0416	3.0938	0.002	**
sex * log(age)	1	0.0075	0.0075	0.0295	1.0337	0.2454	0.406	
residuals	30	0.2178	0.0073	0.8563				
total	33	0.2544						

				arytenoid	cartilage			
sex	1	0.01371	0.0132	0.0233	0.9222	0.1241	0.446	
log(age)	1	0.0829	0.0829	0.1473	5.8104	3.214	0.001	***
sex * log(age)	1	0.0363	0.0363	0.0644	2.5404	2.0199	0.019	*
residuals	30	0.4284	0.0143	0.76079				
total	33	0.5632						

				epiglottis				
sex	1	0.0514	0.0514	0.0609	2.2085	1.8642	0.032	*
log(age)	1	0.0722	0.0722	0.0856	3.1014	2.46638	0.005	**
sex * log(age)	1	0.0249	0.0249	0.0295	1.0685	0.33523	0.362	
residuals	30	0.6985	0.0232	0.8284				
total	33	0.8431						

**Table 5.** Results of the Procrustes ANOVA testing the effects of species, sex and age on thyroid cartilage shape. The table shows the degrees of freedom (Df), sum of squares (SS), mean square (MS), Rsq = Coefficient of determination, F-statistic, *Z*-score and *p*-values for each factor. Statistically significant effects (***p < 0.001) are indicated.

	Df	SS	MS	Rsq	F	Z	Pr(>F)	signif.
				thyroid	cartilage			
species	1	0.06582	0.06582	0.27187	14.4649	3.107	0.001	***
femur	1	0.011704	0.011704	0.04835	2.5722	2.4314	0.006	**
species * femur	1	0.006978	0.006978	0.02882	1.5335	1.3006	0.104	
residuals	30	0.13651	0.00455	0.56387				
total	33	0.242097						

				cricoid	cartilage			
species	1	0.09443	0.094428	0.25195	13.9686	3.9826	0.001	***
femur	1	0.01566	0.015661	0.04179	2.3167	2.0331	0.019	*
species * femur	1	0.01631	0.016311	0.04352	2.4129	2.2237	0.011	*
residuals	30	0.2028	0.00676	0.5411				
total	33	0.37479						

				arytenoid	cartilage			
species	1	0.16542	0.165416	0.27338	13.7605	3.6909	0.001	***
femur	1	0.0271	0.027104	0.04479	2.2547	1.9894	0.024	*
species * femur	1	0.01195	0.011946	0.01974	0.9938	0.2263	0.423	
residuals	30	0.36063	0.012021	0.59601				
total	33	0.60508						

				epiglottis				
species	1	0.12051	0.120512	0.10123	4.1877	2.8732	0.002	**
femur	1	0.0666	0.066596	0.05594	2.3142	1.7262	0.045	*
species * femur	1	0.04942	0.04942	0.04151	1.7173	1.2149	0.115	
residuals	30	0.86333	0.028778	0.72517				
total	33	1.19052						

**Table 6.** Trajectory analysis for five age groups representing stages from neonates to old adults from House mice (MM) and California mice (PC). *Distance* values represent the total length of each trajectory. *Direction* refers to the vector along which shape changes occur in morphospace. *d*, pairwise absolute difference in path distances; *r*, correlation coefficient between angles; UCL, upper confidence level; *Z*, test statistic; *P*, significant effects (*p < 0.05; **p < 0.01).

cartilage	trajectory	MM	PC	*d*/*r*	UCL (95%)	*Z*	*P*/Signif.
thyroid cartilage	distance	0.252	0.207	0.0456	0.078	0.824	0.204
	direction			0.68	36.7	2.35	0.003**
cricoid cartilage	distance	0.221	0.224	0.0031	0.130	−1.647	0.939
	direction			−0.307	125.6	1.11	0.153
arytenoid cartilage	distance	0.668	0.341	0.3276	0.235	2.220	0.01*
	direction			0.471	38.9	2.31	0.013*
epiglottis	distance	0.692	0.545	0.1470	0.2234	0.883	0.211
	direction			−0.386	154.5	0.652	0.429

**Table 7.** Relative eigenvalue dispersion (Vrel) values for House mice (MM) and California mice (PC) for each of the four laryngeal cartilages.

	MM Vrel	PC Vrel	effect size	***p* value**
thyroid cartilage	0.173	0.122	0.767	0.443
cricoid cartilage	0.156	0.072	1.656	0.098
arytenoid cartilage	0.391	0.149	2.455	0.014*
epiglottis	0.176	0.131	0.663	0.507

Thyroid, cricoid and arytenoid cartilages of the California mouse were not sexually dimorphic in shape, but we found that the shape of the epiglottis was sexually dimorphic (table 4). None of the laryngeal cartilages in *Mus musculus* were sexually dimorphic in shape (supplementary material, table S1).

**Figure 4.**
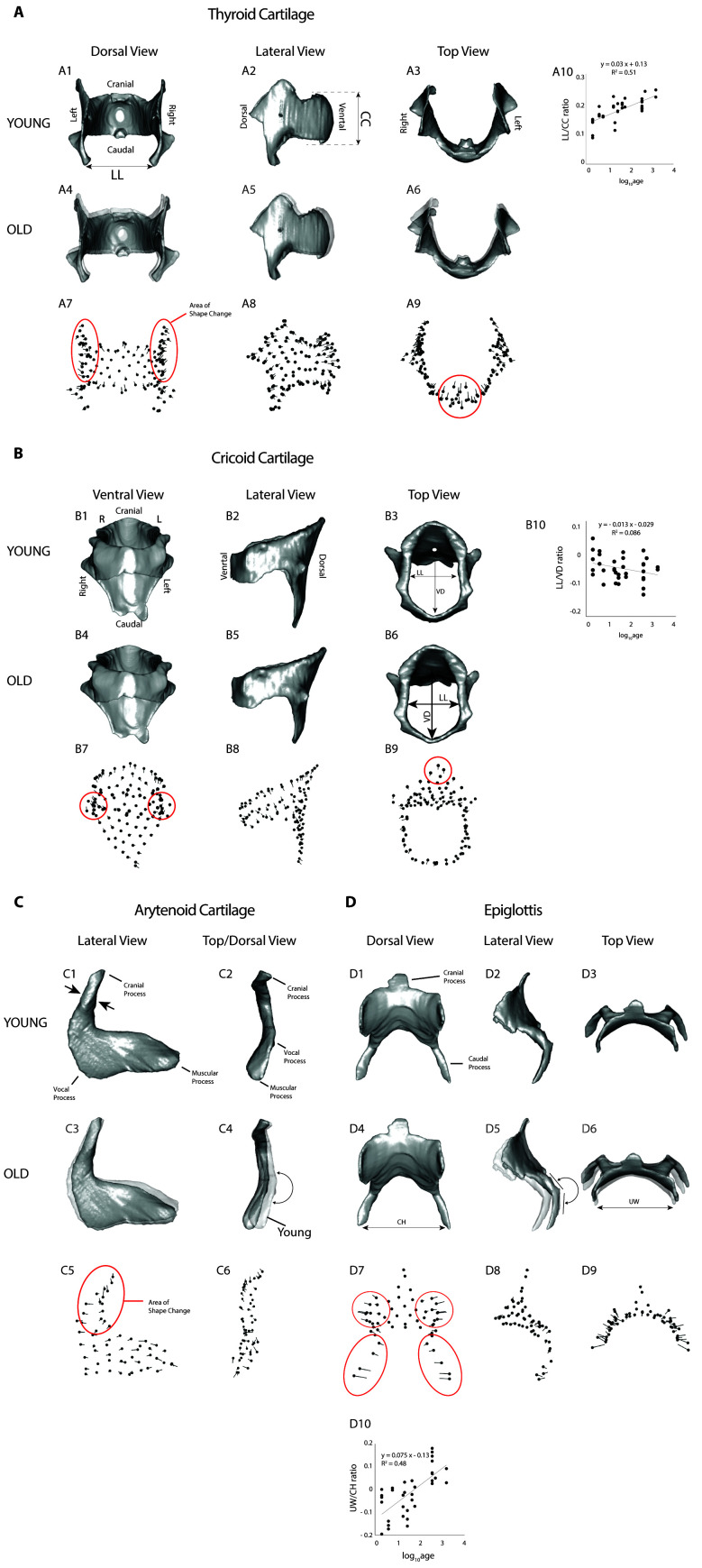
Age-related shape changes of four larynx cartilages in California mice. A: Thyroid cartilage. B: Cricoid cartilage. C: Arytenoid cartilage. D: Epiglottis. A1–A6, B1–B6, C1–C4, D1–D6: Warped cartilage images. A7–A9, B7–B9, C5–C6, D7–D9: Vector plots of total shape change. The warped image of young animals was overlaid onto that of old animals in A4, A5, A6, C3, C4, D5, D6. Each landmark is associated with a vector, and the largest vectors exhibit the greatest size-related change. The direction of the change (from small to large) is indicated by the direction of the vector (away from the landmark). A10, B10, D10: the ratio of two linear measurements taken on the thyroid, the cricoid cartilage and the epiglottis changes over time. Thyroid: LL, latero-lateral width; CC, cranio-caudal height of the thyroid cartilage. Cricoid: VD, dorso-ventral diameters of the airway; LL, latero-lateral diameter of the airway measured at the caudal end of the cricoid cartilage. Epiglottis: UW, cranial wings distance; CH, caudal horns distance.

The thyroid cartilage changed shape with age (table 4) in *P. californicus*. In thyroid cartilages, the areas of greatest shape change are the cranial aspect of the two laminae, and the ventral midline area. Shape differences imply a greater medio-lateral to dorso-ventral width ratio in younger (and smaller) mice than in older (and larger) ones (figure 4). A ‘lollipop’ plot in the third row of figure 4 shows age-related landmark displacement as vectors as an alternative representation of the shape changes. The lateral widening was confirmed by linear measurements (figure 4A10). Both measurements (latero-lateral width and cranio-caudal height; Thyroid LL, Thyroid CC data in the supplementary material) scaled with negative allometry on age but scaling factors were different, and therefore their ratio changed with age. Shape changes resemble those described for House mice (supplementary material, table S1).

Significant age-related shape changes of the cricoid cartilage are subtle in the California mouse (table 4). Older individuals have relatively prominent lateral processes that articulate with the thyroid cartilage and relatively taller dorsal laminae (figure 4B). Two linear measures quantifying the intralaryngeal lumen scaled with negative allometry on age but scaling factors are different, and therefore their ratio changes with age (figure 4B10). In House mice, a significant age-related shape change was also observed for the cricoid cartilage (supplementary material, table S1). Shape changes highlight an interesting species difference in facilitating the interaction between thyroid and cricoid cartilage through the cricothyroid joint. In both mouse species, the thyroid cartilage widens laterally which changes the biomechanics of the thyro-cricoid joint. While in California mice, it is the cricoid cartilage that exhibits shape change (a lateral widening of the processes that support the joint), it is the caudal process of the thyroid cartilage in House mice which alters their angulation to maintain the joint with the cricoid cartilage.

In California mice, the shape of the arytenoid cartilage changes significantly throughout development (table 4). In older mice, two notable changes were observed. First, the dorsal horn narrows. Second, the muscular process and vocal process form a more acute angle when viewed dorsally (figure 4C4). These changes may be influenced by mechanical stress, as both processes serve as attachment sites for several intrinsic muscles, facilitating abduction, adduction and vocal fold length adjustments. These shape changes are similar to those previously described in House mice (supplementary material, table S1).

Two age-related shape changes were observed in the epiglottis of California mice (table 4). First, in older animals, the two caudal processes that connect with the thyroid cartilage move closer together. Second, the lateral wings of the lamina extend further laterally, making the lamina appear flatter (figure 4D). Two linear measurements scale with negative allometry as the mice age, but the scaling factors differ, resulting in a change in their ratio over time (figure 4D10). While the lateral wings of the lamina also extend laterally in House mice, the caudal processes are either very small or absent (supplementary materials, table S1).

### 3.3. Shape and integration of vocal organs in House mice and California mice

**Figure 5.**
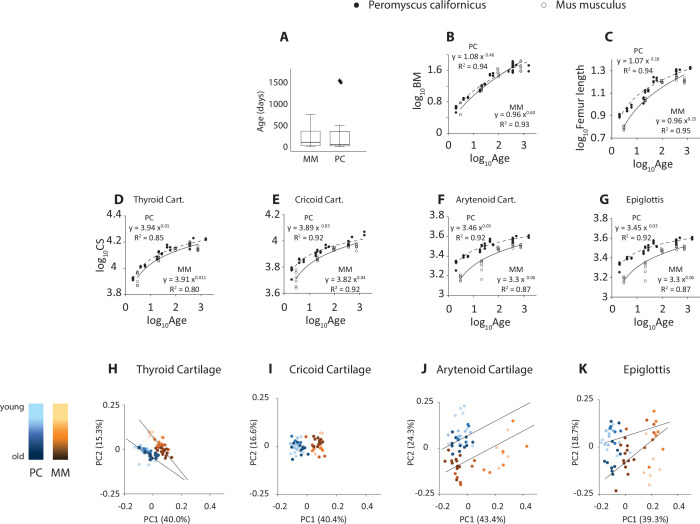
Larynx size and shape of House and California mice. (A) Age ranges of the two datasets. (B**–**G) Relationships between age and body mass, femur length and centroid size (CS) of four cartilages. (*Peromyscus*: dashed line; *Mus*: solid line). (H**–**K): Principal component analysis displaying the first two principal components (PC1 and PC2) of the shape variation derived from the trajectory analysis. The plot highlights major axes of variation, with each point representing an individual observation. Trajectory lines indicate the directional trends in shape change, with five distinct age bins differentiated in the analysis (age group centroids designated by large circle or square). MM, Mus musculus; PC, Peromyscus californicus; CS, centroid size; BM, body mass.

Body masses of the California mouse and House mouse samples were similar, but femur lengths were approximately 15% longer in California mice (figure 5A-C). This suggests that House mice may possess relatively greater lean and fat mass compared with California mice. The centroid sizes of individual cartilages were 15–20% larger in California mice than in House mice, which aligned more closely with the skeletal measurement than with body mass (see figure 5D-G).

Figure 5H through 5K display plots of the first two principal components of shape variation for each cartilage. These plots illustrate that individuals from the two species are consistently separated along PC1 and/or PC2, indicating that interspecific differences in shape are evident across all developmental stages. Linear mixed-effects models (main effects: species and femur length) confirmed that both species identity and skeletal size contributed significantly to shape variation (table 5). Across the four cartilages, species explained 6.6% to 16.5% of the shape variance, while femur length accounted for an additional 1.2% to 6.7%.

**Figure 6.**
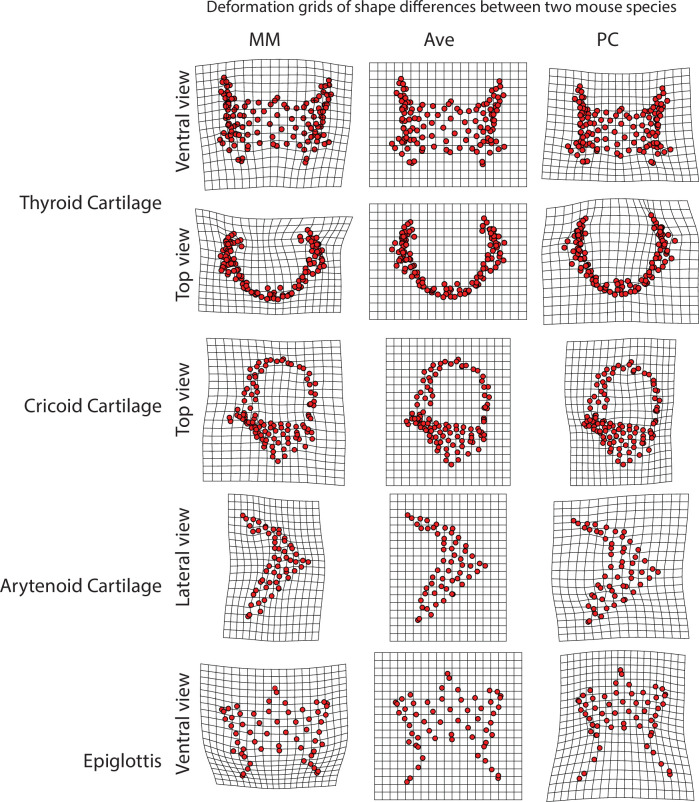
Shape differences of four laryngeal cartilages. Deformation grids showing shape differences between adult House mouse (MM) and California mouse (PC). The deformation of the grid summarizes changes required to perfectly superimpose the landmarks from the adult grand mean (centre panels) onto the landmarks of each the species-specific adult means. Deformations are calculated via the thin plate spline algorithm which creates a smooth mapping by minimizing the bending energy matrix.

While PC1–PC2 plots (e.g. figure 5H) appear to show increased divergence with age, especially in the arytenoid cartilage, these two axes do not fully capture age-related variation, and projections of regression lines in this space may be misleading. Therefore, we utilized high-dimensional multivariate trajectory analysis (table 6). This analysis revealed that the trajectories of the arytenoid and thyroid cartilages differ significantly in direction between species, indicating distinct patterns of shape change during development. By contrast, no significant trajectory differences were detected for the cricoid or epiglottic cartilages. Figure 6 illustrates the shape differences of four laryngeal cartilages in adult representatives from both mouse species.

**Figure 7.**
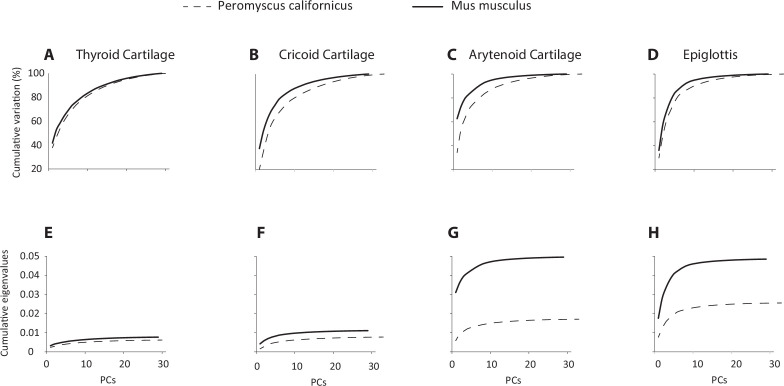
Principal component analysis. (A–D): Cumulative percentage of variation accounted for by each principal component in the species-specific PCAs allows comparison of the distribution of variance in shape space for the two species. (E–H): Cumulative eigenvalues for the species-specific covariance matrices for each cartilage allows comparison of total shape variance between the species.

Finally, we explored trait variability by plotting cumulative eigenvalues and proportion of variance for each shape axis (figure 7). The proportion of variance accounted for on the first few principal components was consistently higher in House mice (figure 6 E-H). Although eigenvalue dispersion values (Vrel) were consistently larger in House mice, the differences were only significant for the arytenoid cartilage (table 7).

## 4. Discussion

This investigation uncovered new insights into the developmental and evolutionary mechanisms shaping vocal anatomy. In both California and House mice, laryngeal cartilage shape was most strongly influenced by size and age, and despite their differences, the shape of vocal organ cartilages was surprisingly similar between the two species.

The scaling results for California mice were comparable with findings in House mice [16,65] with a notable difference in airway morphology. The ventral pouch volume increased with positive allometry during development in California mice, but in House mice, ventral pouch volume in neonates and old individuals was similar [21]. The ventral pouch plays a role in high-frequency whistling of rodents. Its geometry determines the fundamental frequency of the whistles [9,10]. The negative allometry of the laryngeal airway in House mice can explain the spectral overlap between ultrasonic whistles of young, small mice and older, larger mice. A systematic analysis of spectral properties of ultrasonic whistles in California mice is currently unavailable.

Age, but not sex (except for the epiglottis), influences cartilage shape. Developmental shape changes in the thyroid, cricoid, arytenoid and epiglottis cartilages involve age-related shifts in dimensions and joint biomechanics likely influenced by mechanical stress, with many patterns paralleling those seen in House mice [16].

California and House mice had similar body masses but differed in femur length and laryngeal cartilage size. Skeletal size contributes to shape differences across development; however, while developmental trajectories diverged to increase species differences, significant shape changes were mainly observed in the arytenoid cartilage. House mice exhibited greater shape variation concentrated in fewer principal components than California mice, suggesting more constrained shape development likely resulting from reduced genetic diversity and artificial selection in this domesticated strain.

### 4.1. Shape and integration of vocal organs in House mice and California mice

Rodents display a wide range of vocal behaviours [4] and use two distinct vocal production mechanisms [39,40]. Despite the differences in vocal behaviour between House mice and California mice, the cartilaginous framework of the larynx exhibits relatively small shape differences. The variation in vocal behaviour between California and House mice is more likely supported by differences in the neural circuitry controlling vocal movements in these species, and not by large differences in larynx structure. Future studies may explore the role of soft tissue components of the larynx, such as the intrinsic laryngeal muscles and vocal fold lamina propria, in supporting behavioural differences in vocal behaviour.

The relative eigenvalue dispersion ranged from 0.1 to 0.4 (table 7), indicating moderate concentration of variation on the first few eigenvalues, and thus a moderate degree of integration [62,64]. This implies that there is integration, but trait covariation is not strongly canalized within each cartilage. This analysis did not evaluate integration across the different cartilages, however. With the exception of the arytenoid cartilage, the magnitude of integration was comparable between the two species.

**Figure 8.**
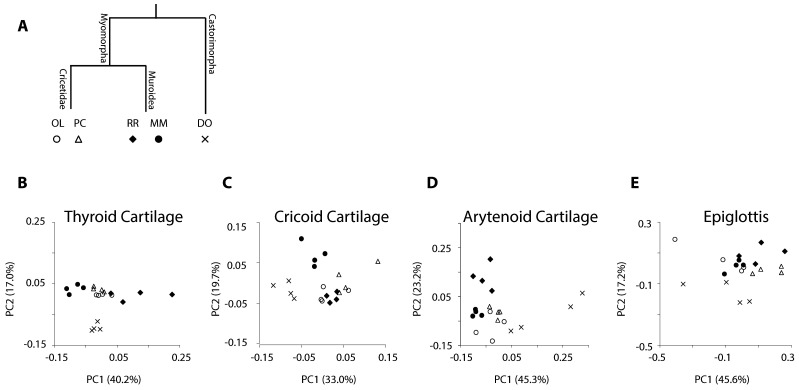
Shape differences of laryngeal cartilages. (A) Rodents are the most species-rich group among mammals. Cricetids (*Onychomys spec*., OL; and *Peromyscus californicus*, PC) and Muroids (*Rattus rattus*, RR; *Mus musculus*, MM) are grouped with Myomorpha. The kangaroo rat (*Dipodomys ordii*, DO) is grouped with Castorimorpha. (B**–**E) Principal component analysis of the Procrustes shape coordinates from landmarks and semilandmarks. Each point represents the cartilage shape of an individual. The first and second principal components (PC) are shown. Note that axis scaling differs between cartilages.

Thus, both laryngeal shape and overall strength of integration are quite similar between the two species despite belonging to phylogenetically distant families. High structural similarity may reflect either a low degree of diversification in laryngeal anatomy or convergence towards a similar structure. The latter has been frequently found between Old World and New World rodents [66,67]. Convergent evolution may have occurred as adaptation to comparable ecological niches or functional demands requiring similar morphologies despite their distant evolutionary relationship. This question remains challenging to resolve for the larynx because few species have been investigated. A previous study comparing four rodent species found that shape differences between two murid rodents (*Mus musculus* and *Rattus norvegicus*) were greater than those between House mice and another cricetid rodent (*Onychomys spec*.) [20]. When the new data from the California mice are incorporated (figure 8), it is clear that shape variation is not phylogenetically structured and the patterns are complex. This argues against simple conservation of morphology, but additional comparative data is necessary to clarify whether functional convergence is a good explanation.

To date, most attention has been given to the soft tissue components of the rodent larynx [39,47,48,68–71]. Overall larynx shape and size have been described for only a few species (*Mus musculus*, *Rattus rattus*, *Pedetes capensis*) [16,20,41,72]. This knowledge gap stands in stark contrast to the comparative morphology of vocal organs available for other vertebrate groups, such as primates, carnivores, artiodactyla and birds (e.g. [73–78]). The limited research on the rodent larynx is unsurprising, given the challenges of quantifying the morphology of a small yet complex organ—a challenge also noted for the small but highly efficient sound source in bats [79]. Without broader sampling, it remains premature to generalize morphological patterns of laryngeal anatomy across rodents.

### 4.2. The form–function relationship of the rodent larynx

Current data suggest that different vocal behaviours in two distantly related rodent species can be supported by similar laryngeal morphology. In the absence of large structural differences of the vocal organ, what other biological functions might be responsible for laryngeal diversification or similarity? The larynx may be influenced by natural selection directly related to its roles in acoustic and respiratory behaviours and via integration with the jaw and pectoral girdle (and thus indirectly by selection on the feeding and locomotor systems). Ecomorphological studies have shown that dietary specializations in rodents are reflected in cranial and dental anatomy [80,81]. Similarly, locomotion patterns are mirrored in the cranial and postcranial anatomy of rodents [82–84]. House mice and California mice share similar diet and locomotion patterns, which may contribute to the observed similarities in laryngeal morphology.

While House mice do not produce long-distance calls, the hallmark long-distance call of cricetid rodents is produced by both sexes to communicate over large distances [85–87]. Long-distance calls must be sufficiently loud to achieve ecologically relevant communication distances and therefore exhibit comparatively high intensities in both sexes [42,88]. Both sexes perform similar roles in foraging, parental care and nest defence [89]. It is therefore less surprising that no sexual dimorphism was found in three main laryngeal cartilages.

Cricetid rodents exhibit two different sound production mechanisms to produce their long-distance calls [16,40,41,70]. The ventral pouch plays a central role in ultrasonic whistle production [9,10], and therefore its large size in cricetid rodents was interpreted as supportive of producing a wide range of fundamental whistle frequencies [70,71]. Whether the ventral pouch also plays a role in the production of low-frequency long-distance calls of California mice is unknown.

### 4.3. Sources of laryngeal shape differences within species—age, sex, size

All components of the mature larynx are already present in the neonate larynx but postnatal age-dependent changes in laryngeal cartilage shape are common in mammals. For example, in human females [17], as well as in House mice [16] and California mice (this study), the angle between the left and right thyroid laminae increases with age. By contrast, this angle decreases with age in human males [17] and in both male and female North American elk (Cervus) [76]. The factors driving the direction of these ontogenetic changes in laryngeal shape remain unclear but are significant, as these shape changes in laryngeal cartilages are associated with the remodelling of the intra-laryngeal airway in humans [90] and House mice [21]. Age-related changes in laryngeal structure can lead to corresponding voice changes or age-related functional impairments in speech or airway protection [91,92].

Hormonal profiles also play a significant role in laryngeal shape variation within species and often contribute to sexual dimorphism in primates [73] and ungulates [15]. In human males, the thyroid cartilage grows in size, and the angle between the laminae decreases with age [17]. In females, postnatal laryngeal shape is influenced by a more complex interaction of age, body mass index (BMI) and height. For example, the angle between the laminae increases with age but remains smaller in females with higher BMI [17]. In many mammals, sexual dimorphism in laryngeal size and shape is typically associated with selective pressures favouring specific acoustic features. For example, a lower fundamental frequency in human males results from a larger larynx that houses almost 50% longer vocal folds than in females [93]. However, no sexual dimorphism in laryngeal size and shape has been observed in California mice (this study), House mice [16], or two other cricetid rodents [71] despite some behavioural differences such as vocal rates that differ between males and females [94]. Further research is needed to clarify these differences. For example, intrinsic laryngeal muscles may display sexually dimorphic fibre patterns [95].

Size differences contributed to the observed shape differences between the two species (table 5). The variation in overall laryngeal shape was linked to a roughly 15% difference in skeletal body size. However, to determine whether size differences truly drive shape variation, a larger sample of adult individuals is needed [96]. Unfortunately, the current sample sizes were insufficient to assess the allometric effects on laryngeal size and shape in adults. California mice are ideal for studying these allometric effects, as they display significant variation in body mass due to environmental factors and different social housing conditions [97,98].

## 5. Conclusions

This study characterized and compared the developmental trajectories of laryngeal cartilages and airways in two rodent species. While age-related effects on laryngeal shape were evident, no sexual dimorphism was detected in either species. Although significant differences in laryngeal shape were observed between the two species, they appear to have converged on similar overall laryngeal anatomy. Trait integration was largely comparable between House mice and California mice, with the exception of one cartilage. These findings highlight the importance of studying species beyond the commonly used House mice to gain a deeper mechanistic understanding of the diversity in vocal behaviour among rodents.

## Data Availability

Surface renditions in stereolithography format of all specimens are available on Morphobank [53], project 5517. Code used to analyze these data is provided upon request. Supplementary material is available online [99].

## References

[1] Cox PG, Hautier L. 2015 Evolution of the rodents: advances in phylogeny, functional morphology and development (no.5). Cambridge, UK: Cambridge University Press.

[2] Wolff JO, Sherman PW. 2007 Rodent societies as model systems. Rodent societies: an ecological and evolutionary perspective, (eds RJ Wolff, PW Sherman), pp. 3–7. Chicago, IL, USA: University of Chicago Press.

[3] Fernández-Vargas M, Riede T, Pasch B. 2022 Mechanisms and constraints underlying acoustic variation in rodents. Anim. Behav. **184**, 135–147. (10.1016/j.anbehav.2021.07.011)

[4] Dent ML, Fay RR, Popper AN (eds). 2018 Rodent bioacoustics. vol. 67. New York, NY, USA: Springer International Publishing.

[5] Müller J. 1847 Űber die bisher unbekannten typischen Verschiedenheiten der Stimmorgane der Passerinen. Abh. K. Akad. Wiss. Berlin 71.

[6] Ames PL. 1971 The morphology of the syrinx in passerine birds. New Haven, CT, USA: Peabody Museum of Natural History, Yale University.

[7] Riede T, Goller F. 2014 Morphological basis for the evolution of acoustic diversity in oscine songbirds. Proc. R. Soc. B Biol. Sci. **281**, 20132306. (10.1098/rspb.2013.2306) PMC3924064 24500163

[8] Garcia SM, Kopuchian C, Mindlin GB, Fuxjager MJ, Tubaro PL, Goller F. 2017 Evolution of vocal diversity through morphological adaptation without vocal learning or complex neural control. Curr. Biol. **27**, 2677–2683.(10.1016/j.cub.2017.07.059) 28867206 PMC5599221

[9] Riede T, Borgard HL, Pasch B. 2017 Laryngeal airway reconstruction indicates that rodent ultrasonic vocalizations are produced by an edge-tone mechanism. R. Soc. Open Sci. **4**, 170976. (10.1098/rsos.170976) 29291091 PMC5717665

[10] Abhirami S, Agarwalla S, Bhattacharya A, Bandyopadhyay S. 2023 Contribution of the ventral pouch in the production of mouse ultrasonic vocalizations. Phys. Rev. E **107**, 024412. (10.1103/physreve.107.024412) 36932582

[11] Negus VE. 1949 The comparative anatomy and physiology of the larynx. New York, NY, USA: Grune & Stratton, Inc.

[12] Brualla NLM *et al*. 2024 Comparative anatomy of the vocal apparatus in bats and implications for the diversity of laryngeal echolocation. Zool. J. Linn. Soc **202**, d180. (10.1093/zoolinnean/zlad180)

[13] Schneider R. 1964 Der larynx der Säugetiere. Handbuch der Zool. **5**, 1–128.

[14] Jotz GP, Stefani MA, Pereira da Costa Filho O, Malysz T, Soster PR, Leão HZ. 2014 A morphometric study of the larynx. J. Voice **28**, 668–672. (10.1016/j.jvoice.2014.03.008) 24814367

[15] Frey R, Riede T. 2003 Sexual dimorphism of the larynx of the Mongolian gazelle (Procapra gutturosa Pallas, 1777) (Mammalia, Artiodactyla, Bovidae). Zool. Anz. J. Comp. Zool **242**, 33–62. (10.1078/0044-5231-00086)

[16] Riede T, Coyne M, Tafoya B, Baab KL. 2020 Postnatal development of the mouse larynx: negative allometry, age-dependent shape changes, morphological integration, and a size-dependent spectral feature. J. Speech Lang. Hear. Res. **63**, 2680–2694. (10.1044/2020_jslhr-20-00070) 32762490

[17] Riede T, Stein A, Baab KL, Hoxworth JM. 2023 Post-pubertal developmental trajectories of laryngeal shape and size in humans. Sci. Rep. **13**, 7673. (10.1038/s41598-023-34347-w) 37169811 PMC10175495

[18] Titze IR. 1989 Physiologic and acoustic differences between male and female voices. J. Acoust. Soc. Am. **85**, 1699–1707. (10.1121/1.397959) 2708686

[19] Unteregger F, Honegger F, Potthast S, Zwicky S, Schiwowa J, Storck C. 2017 3D analysis of the movements of the laryngeal cartilages during singing. Laryngoscope **127**, 1639–1643. (10.1002/lary.26430) 27882556

[20] Borgard HL, Baab K, Pasch B, Riede T. 2020 The shape of sound: a geometric morphometrics approach to laryngeal functional morphology. J. Mamm. Evol. **27**, 577–590. (10.1007/s10914-019-09466-9)

[21] Darwaiz T, Pasch B, Riede T. 2022 Postnatal remodeling of the laryngeal airway removes body size dependency of spectral features for ultrasonic whistling in laboratory mice. J. Zool. **318**, 114–126. (10.1111/jzo.13003)

[22] Shorter KR, Crossland JP, Webb D, Szalai G, Felder MR, Vrana PB. 2012 Peromyscus as a mammalian epigenetic model. Genet. Res. Int. **2012**, 1–11. (10.1155/2012/179159) PMC3335729 22567379

[23] Bedford NL, Hoekstra HE. 2015 Peromyscus mice as a model for studying natural variation. eLife **4**, e06813. (10.7554/elife.06813) 26083802 PMC4470249

[24] Havighorst A, Crossland J, Kiaris H. 2017 Peromyscus as a model of human disease. Semin. Cell Dev. Biol. **61**, 150–155. (10.1016/j.semcdb.2016.06.020) 27375227

[25] Chambers LK, Singleton GR, Krebs CJ. 2000 Movements and social organization of wild house mice (Mus domesticus) in the wheatlands of northwestern Victoria, Australia. J. Mammal. **81**, 59–69.

[26] Ribble DO. 1991 The monogamous mating system of Peromyscus californicus as revealed by DNA fingerprinting. Behav. Ecol. Sociobiol. **29**, 161–166.

[27] Ribble DO, Salvioni M. 1990 Social organization and nest co-occupancy in Peromyscus californicus, a monogamous rodent. Behav. Ecol. Sociobiol. **26**, 9–15.

[28] Ribble DO. 2003 The evolution of social and reproductive monogamy in Peromyscus: evidence from Peromyscus californicus (the California mouse). In Monogamy: mating strategies and partnerships in birds, humans, and other mammals (eds U Reichard, C Boesh), pp. 81–92. Cambridge, UK: Cambridge University Press.

[29] Gubernick DJ, Alberts JR. 1987 The biparental care system of the California mouse, Peromyscus californicus. J. Comp. Psychol. **101**, 169. 3608423

[30] Cantoni D, Brown RE. 1997 Paternal investment and reproductive success in the California mouse, Peromyscus californicus. Anim. Behav. **54**, 377–386. 9268470 10.1006/anbe.1996.0583

[31] Gootwine E, Spencer TE, Bazer FW. 2007 Litter-size-dependent intrauterine growth restriction in sheep. Animal **1**, 547–564. (10.1017/s1751731107691897) 22444412

[32] Rödel HG, Prager G, Stefanski V, von Holst D, Hudson R. 2008 Separating maternal and litter-size effects on early postnatal growth in two species of altricial small mammals. Physiol. Behav. **93**, 826–834. (10.1016/j.physbeh.2007.11.047) 18187167

[33] Aldinger KA, Sokoloff G, Rosenberg DM, Palmer AA, Millen KJ. 2009 Genetic variation and population substructure in outbred CD-1 mice: implications for genome-wide association studies. PLoS One **4**, e4729. (10.1371/journal.pone.0004729) 19266100 PMC2649211

[34] Havighorst A, Kaza V, Kiaris H. 2019 The peromyscus genetic stock center. In The biological resources of model organisms (eds RL Jarret, K McCluskey), pp. 183–196, 1st edn. Boca Raton, FL, USA: CRC Press.

[35] Kalcounis-Rueppell MC, Petric R, Briggs JR, Carney C, Marshall MM, Willse JT, Rueppell O, Ribble DO, Crossland JP. 2010 Differences in ultrasonic vocalizations between wild and laboratory California mice (Peromyscus californicus). PLoS One **5**, e9705. (10.1371/journal.pone.0009705) 20368980 PMC2848568

[36] Johnson SA *et al*. 2017 Characterization of vocalizations emitted in isolation by California mouse (Peromyscus californicus) pups throughout the postnatal period. J. Comp. Psychol. **131**, 30–39. (10.1037/com0000057) 28182483 PMC5310764

[37] Rieger NS, Marler CA. 2018 The function of ultrasonic vocalizations during territorial defence by pair-bonded male and female California mice. Anim. Behav. **135**, 97–108. (10.1016/j.anbehav.2017.11.008)

[38] Musolf K, Hoffmann F, Penn DJ. 2010 Ultrasonic courtship vocalizations in wild house mice, Mus musculus musculus. Anim. Behav. **79**, 757–764.

[39] Roberts LH. 1975 The functional anatomy of the rodent larynx in relation to audible and ultrasonic cry production. Zool. J. Linn. Soc. **56**, 255–264. (10.1111/j.1096-3642.1975.tb00268.x)

[40] Riede T, Kobrina A, Bone L, Darwaiz T, Pasch B. 2022 Mechanisms of sound production in deer mice (Peromyscus). J. Exp. Biol. **225**, b243695. (10.1242/jeb.243695) PMC9163445 35413125

[41] Riede T, Kobrina A, Pasch B. 2024 Anatomy and mechanisms of vocal production in harvest mice. J. Exp. Biol **227**, jeb246553. (10.1242/jeb.246553) 38269528 PMC11698031

[42] Brzozowski R, Kobrina A, Mahoney SM, Pasch B. 2023 Advertising and receiving from heights increases transmission of vocalizations in semi-arboreal mice. Behav. Ecol. Sociobiol. **77**, 1–10. (10.1007/s00265-023-03352-4)

[43] Riede T. 2011 Subglottal pressure, tracheal airflow, and intrinsic laryngeal muscle activity during rat ultrasound vocalization. J. Neurophysiol. **106**, 2580–2592. 21832032 10.1152/jn.00478.2011 PMC3214115

[44] Riede T. 2013 Stereotypic laryngeal and respiratory motor patterns generate different call types in rat ultrasound vocalization. J. Exp. Zool. A Ecol. Genet. Physiol. **319**, 213–224. 23423862 10.1002/jez.1785 PMC3926509

[45] Lawrence BD, Simmons JA. 1982 Measurements of atmospheric attenuation at ultrasonic frequencies and the significance for echolocation by bats. J. Acoust. Soc. Am. **71**, 585–590. (10.1121/1.387529) 7085967

[46] Steppan SJ, Schenk JJ. 2017 Muroid rodent phylogenetics: 900-species tree reveals increasing diversification rates. PLoS One **12**, e0183070. (10.1371/journal.pone.0183070) 28813483 PMC5559066

[47] Tabler JM *et al*. 2017 Cilia-mediated hedgehog signaling controls form and function in the mammalian larynx. eLife **6**, e19153. (10.7554/elife.19153) 28177282 PMC5358977

[48] Lungova V, Thibeault SL. 2020 Mechanisms of larynx and vocal fold development and pathogenesis. Cell. Mol. Life Sci. **77**, 3781–3795. (10.1007/s00018-020-03506-x) 32253462 PMC7511430

[49] Cheverud JM. 1996 Developmental integration and the evolution of pleiotropy. Am. Zool. **36**, 44–50.

[50] Hallgrímsson B, Willmore K, Hall BK. 2002 Canalization, developmental stability, and morphological integration in primate limbs. Am. J. Phys. Anthropol. **119**, 131–158. (10.1002/ajpa.10182) PMC5217179 12653311

[51] Sikes RS, Animal Care and Use Committee of the American Society of Mammalogists. 2016 Guidelines of the American society of mammalogists for the use of wild mammals in research and education. J. Mammal. **97**, 663–688. (10.1093/jmammal/gyw078) 29692469 PMC5909806

[52] Sanger TJ, Norgard EA, Pletscher LS, Bevilacqua M, Brooks VR, Sandell LJ, Cheverud JM. 2011 Developmental and genetic origins of murine long bone length variation. J. Exp. Zool. B **316**, 146–161. (10.1002/jez.b.21388) PMC3160521 21328530

[53] O’Leary MA, Kaufman SG. 2012 MorphoBank 3.0: web application for morphological phylogenetics and taxonomy. See http://www.morphobank.org.

[54] Core R. 2015 Team. R: a language and environment for statistical computing, 2021.

[55] Gower JC. 1975 Generalized procrustes analysis. Psychometrika **40**, 33–51.

[56] Rohlf FJ, Slice D. 1990 Extensions of the procrustes method for the optimal superimposition of landmarks. Syst. Zool. **39**, 40–59. (10.2307/2992207)

[57] Zelditch ML, Lundrigan BL, Garland T. 2004 Developmental regulation of skull morphology. I. Ontogenetic dynamics of variance. Evol. Dev. **6**, 194–206. (10.1111/j.1525-142x.2004.04025.x) 15099307

[58] Titze I, Riede T, Mau T. 2016 Predicting achievable fundamental frequency ranges in vocalization across species. PLoS Comput. Biol. **12**, e1004907. (10.1371/journal.pcbi.1004907) 27309543 PMC4911068

[59] Schindelin J *et al*. 2012 Fiji: an open-source platform for biological-image analysis. Nat. Methods **9**, 676–682. (10.1038/nmeth.2019) 22743772 PMC3855844

[60] Adams DC, Collyer ML. 2018 Multivariate phylogenetic comparative methods: evaluations, comparisons, and recommendations. Syst. Biol. **67**, 14–31. (10.1093/sysbio/syx055) 28633306

[61] Adams DC, Collyer ML. 2007 The analysis of character divergence along environmental gradients and other covariates. Evolution (N Y) **61**, 510–515. (10.1111/j.1558-5646.2007.00063.x) 17348916

[62] Conaway MA, Adams DC. 2022 An effect size for comparing the strength of morphological integration across studies. Evolution **76**, 2244–2259. (10.1111/evo.14595) 35971251 PMC9804739

[63] Adams DC, Collyer ML, Kaliontzopoulou A, Baken EK. 2002 Geomorph: software for geometric morphometric analysis. R package version 4.0.3.

[64] Watanabe J. 2022 Statistics of eigenvalue dispersion indices: quantifying the magnitude of phenotypic integration. Evolution **76**, 4–28. (10.1111/evo.14382) 34679186

[65] River C. CD-1® IGS Mouse Details. See https://www.criver.com/products-services/find-model/cd-1r-igs-mouse?region=3611.

[66] Ben-Moshe A, Dayan T. 2001 Convergence in morphological patterns and community organization between old and new world rodent guilds. Am. Nat. **158**, 484. (10.2307/3079290) 18707303

[67] Kay EH, Hoekstra HE. 2008 Rodents. Curr. Biol. **18**, R406–R410. (10.1016/j.cub.2008.03.019) 18492466

[68] Inagi K, Schultz E, Ford CN. 1998 An anatomic study of the rat larynx: establishing the rat model for neuromuscular function. Otolaryngol. Head Neck Surg. **118**, 74–81. (10.1016/S0194-5998(98)70378-X) 9450832

[69] Alli O, Berzofsky C, Sharma S, Pitman MJ. 2013 Development of the rat larynx: A histological study. Laryngoscope **123**, 3093–3098. (10.1002/lary.24145) 23918405

[70] Pasch B, Tokuda IT, Riede T. 2017 Grasshopper mice employ distinct vocal production mechanisms in different social contexts. Proc. R. Soc. B Biol. Sci. **284**, 20171158. (10.1098/rspb.2017.1158) PMC5543235 28724740

[71] Smith SK, Burkhard TT, Phelps SM. 2021 A comparative characterization of laryngeal anatomy in the singing mouse. J. Anat. **238**, 308–320. (10.1111/joa.13315) 32996145 PMC7812124

[72] Kupper W. 1970 Der Kehlkopf des afrikanischen Springhasen, Pedetes capensis (Forster 1778) (Mammalia, Rodentia). Z. Fuer Wiss. Zool. **181**, 140–178.

[73] Starck D, Schneider R. 1960 Respirationsorgane, Larynx. Primatologia **3**, 423–587.

[74] Hast MH. 1989 The larynx of roaring and non-roaring cats. J. Anat. **163**, 117–121. 2606766 PMC1256521

[75] Frey R, Gebler A, Fritsch G. 2006 Arctic roars – laryngeal anatomy and vocalization of the muskox (Ovibos moschatus Zimmermann, 1780, Bovidae). J. Zool. **268**, 433–448. (10.1111/j.1469-7998.2006.00053.x)

[76] Frey R, Riede T. 2013 The anatomy of vocal divergence in North American Elk and European red deer. J. Morphol. **274**, 307–319. (10.1002/jmor.20092) 23225193 PMC3928815

[77] Bowling DL *et al*. 2020 Rapid evolution of the primate larynx? PLoS Biol. **18**, e3000764. (10.1371/journal.pbio.3000764) 32780733 PMC7418954

[78] King AS. 1989 Functional anatomy of the syrinx. In Form and function in birds (eds AS King, J McLelland), pp. 105–192, vol. 4. London, UK: Academic Press.

[79] Brualla NLM, Wilson LAB, Doube M, Carter RT, McElligott AG, Koyabu D. 2023 The vocal apparatus: an understudied tool to reconstruct the evolutionary history of echolocation in bats? J. Mamm. Evol. **30**, 79–94. (10.1007/s10914-022-09647-z)

[80] Cox PG, Rayfield EJ, Fagan MJ, Herrel A, Pataky TC, Jeffery N. 2012 Functional evolution of the feeding system in rodents. PLoS One **7**, e36299. (10.1371/journal.pone.0036299) 22558427 PMC3338682

[81] Samuels JX. 2009 Cranial morphology and dietary habits of rodents. Zool. J. Linn. Soc. **156**, 864–888. (10.1111/j.1096-3642.2009.00502.x)

[82] Carrizo LV, Tulli MJ, Dos Santos DA, Abdala V. 2014 Interplay between postcranial morphology and locomotor types in Neotropical sigmodontine rodents. J. Anat. **224**, 469–481. (10.1111/joa.12152) 24372154 PMC4098680

[83] Verde Arregoitia LD, Fisher DO, Schweizer M. 2017 Morphology captures diet and locomotor types in rodents. R. Soc. Open Sci. **4**, 160957. (10.1098/rsos.160957) 28280593 PMC5319359

[84] Woldt KM, Pratt RB, Statham MJ, Barthman‐Thompson LM, Sustaita D. 2024 Comparative skeletal anatomy of salt marsh and western harvest mice in relation to locomotor ecology. J. Anat. **245**, 289–302. (10.1111/joa.14047) 38613221 PMC11259749

[85] Miller JR, Engstrom MD. 2007 Vocal stereotypy and singing behavior in Baiomyine mice. J. Mammal. **88**, 1447–1465. (10.1644/06-mamm-a-386r.1)

[86] Miller JR, Engstrom MD. 2010 Stereotypic vocalizations in harvest mice (Reithrodontomys): harmonic structure contains prominent and distinctive audible, ultrasonic, and non-linear elements. J. Acoust. Soc. Am. **128**, 1501–1510. (10.1121/1.3455855) 20815485

[87] Miller JR, Engstrom MD. 2012 Vocal stereotypy in the rodent genera Peromyscus and Onychomys (Neotominae): taxonomic signature and call design. Bioacoustics **21**, 193–213. (10.1080/09524622.2012.675176)

[88] Green DM, Mull N, Scolman T, Griffiths G, Pasch B. 2020 Active space of grasshopper mouse vocalizations (Onychomys) in relation to woody plant encroachment. Behaviour **157**, 1211–1229. (10.1163/1568539x-bja10046)

[89] Rieger NS, Stanton EH, Marler CA. 2019 Division of labour in territorial defence and pup retrieval by pair-bonded California mice, Peromyscus californicus. Anim. Behav. **156**, 67–78. (10.1016/j.anbehav.2019.05.023)

[90] Holzki J, Brown KA, Carroll RG, Coté CJ. 2018 The anatomy of the pediatric airway: has our knowledge changed in 120 years? A review of historic and recent investigations of the anatomy of the pediatric larynx. Pediatr. Anesth. **28**, 13–22. (10.1111/pan.13281) 29148119

[91] Martins RHG, Gonçalvez TM, Pessin ABB, Branco A. 2014 Aging voice: presbyphonia. Aging Clin. Exp. Res. **26**, 1–5. (10.1007/s40520-013-0143-5) 24068559

[92] Mallick AS, Garas G, McGlashan J. 2019 Presbylaryngis: a state-of-the-art review. Curr. Opin. Otolaryngol. Head Neck Surg **27**, 168–177. (10.1097/MOO.0000000000000540) 30920986

[93] Puts DA *et al*. 2016 Sexual selection on male vocal fundamental frequency in humans and other anthropoids. Proc. R. Soc. B Biol. Sci. **283**, 20152830. (10.1098/rspb.2015.2830) PMC4855375 27122553

[94] Vieira ML, Brown RE. 2002 Ultrasonic vocalizations and ontogenetic development in California mice (Peromyscus californicus). Behav. Process. **59**, 147–156. (10.1016/s0376-6357(02)00089-x) 12270517

[95] Hoh JFY. 2005 Laryngeal muscle fibre types. Acta Physiol. Scand. **183**, 133–149. (10.1111/j.1365-201X.2004.01402.x) 15676055

[96] Klingenberg CP. 2016 Size, shape, and form: concepts of allometry in geometric morphometrics. Dev. Genes Evol. **226**, 113–137. (10.1007/s00427-016-0539-2) 27038023 PMC4896994

[97] Zhao M, Garland T, Chappell MA, Andrew JR, Harris BN, Saltzman W. 2017 Effects of a physical and energetic challenge on male California mice (Peromyscus californicus): modulation by reproductive condition. J. Exp. Biol. **221**, b168559. (10.1242/jeb.168559) PMC5818025 29170256

[98] Andrew JR, Garland T, Chappell MA, Zhao M, Saltzman W. 2019 Effects of short- and long-term cold acclimation on morphology, physiology, and exercise performance of California mice (Peromyscus californicus): potential modulation by fatherhood. J. Comp. Physiol. B **189**, 471–487. (10.1007/s00360-019-01219-7) 31073767 PMC6667301

[99] Sherwood M, Kaup M, Baab KL, Riede T. 2025 Supplementary material from: Developmental Trajectories in Laryngeal Anatomy of California Mice (Peromyscus californicus). Figshare. (10.6084/m9.figshare.c.8022526) PMC12505160 41069643

